# Biomonitoring of Trace Elements in Subjects Living Near a Hazardous Waste Incinerator: Concentrations in Autopsy Tissues

**DOI:** 10.3390/toxics8010011

**Published:** 2020-02-11

**Authors:** Francisco García, Montse Marquès, Eneko Barbería, Pilar Torralba, Inés Landin, Carlos Laguna, José L. Domingo, Martí Nadal

**Affiliations:** 1 Laboratory of Toxicology and Environmental Health, School of Medicine, IISPV, Universitat Rovira i Virgili, Sant Llorenç 21, 43201 Reus, Catalonia, Spain; francisco.garcia@urv.cat (F.G.); montserrat.marques@urv.cat (M.M.); joseluis.domingo@urv.cat (J.L.D.); 2 Institut de Medicina Legal i Ciències Forenses, Divisió de Tarragona, Rambla del President Lluís Companys 10, 43005 Tarragona, Catalonia, Spain; eneko.barberia@urv.cat (E.B.); mariapilar.torralba@xij.gencat.cat (P.T.); mariaines.landin@urv.cat (I.L.); carlosjavier.laguna@xij.gencat.cat (C.L.)

**Keywords:** trace elements, autopsy tissues, hazardous waste incinerator, temporal trends

## Abstract

The only hazardous waste incinerator (HWI) in Spain started to operate in 1999. Twenty years later, the levels of 11 trace elements (As, Be, Cd, Cr, Hg, Mn, Ni, Pb, Sn, Tl and V) were analyzed in five different autopsy tissues (kidney, liver, brain, bone and lung) from 20 individuals who had been living near the facility. In 2019, As, Be, Tl and V were not detected in any of the analyzed tissues, while Hg could be only quantified in very few samples. The highest levels of Cd and Pb were found in kidney and bone, respectively, while those of Mn were observed in liver and kidney. In turn, the mean concentrations of Cr and Sn were very similar in all tissues. A consistent temporal trend (1998–2019) was only found for Cr and Pb. On the one hand, the mean Cr concentrations in kidney and bone have increased progressively since 1998. In contrast, the mean levels of Pb decreased significantly over time, probably due to ban of Pb as gasoline additive. The data global analysis indicates that the emissions of trace elements by the HWI have not increased the exposure and/or accumulation of these elements in individuals living near the facility.

## 1. Introduction

In 2016, the total waste generated in the EU-28 by all economic activities and households amounted to 2538 million tons, being 100.7 million tons classified as hazardous waste (HW), which means 4% of the total [1]. According to EU statistics, in the period 2010–2016 there was an increasing trend of around 5% in the generation of HW in the EU-28. Although landfilling is still the most predominant practice used to manage HW in the EU, recycling is also relatively important, as up to 37.8% of the total amount was recycled. Other alternatives include backfilling and incineration, either with or without energy recovery. The percentages of HW that is incinerated vary across the EU, with Norway, Denmark and Portugal presenting the highest rates (34%, 19% and 12%). In contrast, the contribution of incineration to HW management in countries, such as Malta, Greece or Bulgaria is nominal.

In 2016, only 3.6% of the HW generated in Spain was incinerated. Currently, Spain counts with only one HW incinerator (HWI), which is located in Constantí (Tarragona, Catalonia, Spain). This facility has been continuously operating for 20 years since 1999, when it started its regular operations. In the 1996–1998 period, a pre-operational surveillance program was conducted to assess potential temporal changes that could occur regarding the exposure to environmental pollutants potentially emitted by the plant. The baseline survey included the analysis of polychlorinated dibenzo-*p*-dioxins and dibenzofurans (PCDD/Fs) and a number of trace elements, two groups of pollutants of high concern for the population, and whose levels in stack air must be periodically controlled [2]. The contents of PCDD/Fs and trace elements were determined in a wide range of environmental and biological samples [3,4,5,6,7,8,9]. Because diet is the most important route of exposure to these chemicals [10,11,12], the dietary intake by the local population was also evaluated [13,14]. The biomonitoring was based on the analysis of 11 trace elements in samples of hair from schoolchildren, blood from general population, as well as samples of autopsy tissues from individuals who had been residing near the plant for at least the last 10 years [3,4,5]. Since then, the concentrations of the same chemicals in the same matrices have been updated every 5 years [15,16,17]. Recently, we have reported the levels of As and a number of metals in human hair and blood of the population living in the neighborhood of the HWI [18,19].

In the present study, the concentrations of trace elements were determined in samples of autopsy tissues collected in 2019 from subjects who had been living near the HWI of Constantí. The temporal trends in the pollutant levels were also established by comparing the current results with those found in the baseline (1998) and the previously performed (2002–2007–2013) studies. Finally, the concentrations of these elements in autopsy tissues were correlated with those found in other biomonitors (human hair and blood) from non-occupationally exposed individuals living in the same area.

## 2. Materials and Methods

### 2.1. Sampling

In 2019, autopsy tissue samples were collected from 20 individuals who—at the time of death—had been residing near the Constantí HWI at least during the previous 10 years. These individuals were not occupationally exposed, being five of them smokers. The samples were collected in close collaboration with forensic doctors from the Tarragona Division of the Institute of Legal Medicine and Forensic Sciences of Catalonia. Samples were obtained from 19 men and one woman, with an average age of 56 years. From each subject, samples (1 g) of the following tissues were obtained: kidney, liver, brain, bone tissue, and lung. One hundred samples (five tissues per individual) were collected. Autopsies and sample collection were performed within the first 24 h after the death. Samples were stored in hermetically sealed polyethylene containers and frozen at −20 °C for processing [20,21,22]. The protocol of the biological surveillance program, number 07/2017, was reviewed and approved by the Ethical Committee for Clinical Research (CEIm) of the Pere Virgili Health Research Institute (IISPV), Reus/Tarragona, Spain, in 20 March 2017. Furthermore, the specific protocol for the biomonitoring study of autopsy tissues, number PR164/19, was complementarily evaluated and approved by the Clinical Research Ethics Committee (CEIC) of the Bellvitge University Hospital, Barcelona, Spain, in 9 May 2019.

### 2.2. Chemical Analysis

The concentrations of arsenic (As), beryllium (Be), cadmium (Cd), chromium (Cr), mercury (Hg), manganese (Mn), nickel (Ni), lead (Pb), tin (Sn), thallium (Tl) and vanadium (V) were determined in all samples. In order to obtain fully comparable data, the same experimental procedure as that used in previous studies of the surveillance program [21,22], was followed. Briefly, 0.5 g of each sample were treated in Teflon vessels containing 5 mL of nitric acid (65%, Suprapur, E. Merck, Darmstadt, Germany) during 8 h at room temperature. Afterwards, samples were heated for 8 h more at 80 °C. After cooling, samples were filtrated, made up to 25 mL with deionized water and properly stored −20 °C until the chemical determination. The analysis of the 11 trace elements was conducted by means of inductively coupled plasma-mass spectrometry (ICP-MS) using an Elan 6000 instrument (Perkin Elmer, Waltham, MA, USA). The limits of detection (LODs) were 0.025 μg/g for Cd, Pb and Tl; 0.05 μg/g for Mn; 0.10 μg/g for As, Be and Hg; 0.25 μg/g for Ni; and 0.50 μg/g for Cr, Sn and V. The quality of the experimental procedure was controlled and assured by analyzing reference materials (Lobster Hepatopancreas, TORT 2, NRC Canada, Ottawa, ON, Canada) and blanks in every batch of samples Reproducibility was assured as reported in previous investigations [22]. Recovery percentages ranged between 84% and 121%.

### 2.3. Statistics

When any of the analyzed metals could not be detected, a concentration equivalent to one-half of their respective detection limit was assumed (ND = 1/2 LOD). Statistical significance of the results was first assessed by applying the Levene test to verify the homoscedasticity of the data. Depending on whether the variance followed a normal distribution or not, a variance analysis (ANOVA) or the Mann-Whitney U-test was subsequently performed. A probability lower than 0.05 (*p* <0.05) was considered significant. Pearson correlations in the concentrations among the five evaluated tissues, as well as with those of human hair and blood [18,19], were also carried out.

## 3. Results

Data regarding the concentrations of trace elements in samples of autopsy tissues collected in 2019 are summarized in Table 1. The levels of As, Be, Tl and V were below their respective detection limits (<0.10, <0.10, <0.025 and <0.50 µg/g, respectively) in all tissues. Mercury could be only detected in one sample of liver and three of kidney, with its levels being under the detection limit in the remaining 96 samples. Cadmium showed the highest levels in kidney, with a mean concentration of 11.10 µg/g, while in other organs the concentrations were much lower (range: 0.02–0.76 µg/g). The average Cr concentration in the five analyzed tissues was very similar, with values ranging from 0.29 to 1.02 μg/g. In turn, Mn could be quantified in all tissues, with relatively higher levels in liver and kidney (1.16 and 0.82 µg/g, respectively). Lead showed higher values in bone (mean: 1.00 µg/g; range: <0.025–5.39 µg/g) than in the other evaluated tissues. Finally, Sn was also detected in the five tissues, with similar levels in all (range: 0.78–1.94 µg/g).

Table 2 shows the concentrations of the metals analyzed in autopsy samples (brain, bone, kidney, liver, and lung) in the 1998 (baseline), 2003, 2007, 2013, and 2019 surveys, as well as the percentage variation in the periods 1998–2019 and 2013–2019. Between 1998 and 2019, a significant decrease in Cd, Hg and Pb concentrations was noticed (20%, 70% and 91%, respectively), while only Cd showed a significant reduction between the campaigns performed in 2013 and 2019 (45%; *p* < 0.001).

In kidney, a significant increase in Cr levels, as well as in those of Hg and Sn, was observed between the baseline (1998) and the current (2019) studies. In contrast, none of the evaluated elements showed a significant change of concentration between 2013 and 2019. In 2019, the mean concentrations of As, Be, Hg, Tl and V in brain were lower than their respective detection limits. Regarding Cr, a non-significant decrease (44%) was observed in the period 2013–2019, while a non-significant increase was noted between 1998 and 2019 (*p* > 0.05). Lead was the only element for which a significant change in concentration was observed in kidney, with a significant reduction (94%; *p* < 0.001) between the baseline (1998) and the current (2019) surveys.

In bone, most elements showed a decrease of their concentrations with respect to those found in the baseline study, with significant reductions in Cd, Pb and Sn (50%, 75% and 74%, respectively). In contrast, Cr showed a significant increase, from 0.51 to 1.02 µg/g, between 1998 and 2019. With respect to the previous survey, conducted in 2013, the levels of Sn significantly increased (*p* < 0.001) and those of Mn significantly decreased (*p* < 0.05).

In lung, most trace elements presented a reduction of their concentration over time. However, the difference between 1998 and 2019 were only significant for Pb (*p* < 0.001). In contrast, Ni levels significantly increased (88%; *p* < 0.01). With respect to our most recent study (2013), none of the trace elements showed an increase of concentration in lung, while significant decreases were noted for Cr and Mn (*p* < 0.05 and *p* < 0.01, respectively).

Some fluctuations in the concentrations of trace elements in the analyzed human tissues were found. However, a general trend was not observed after 20 years of continuous operation of the plant. When considering the whole assessment period (1998–2019), no changes were observed for any metal, excepting Cr and Pb. On one hand, between 1998 and 2019, Cr concentration increased in all tissues excepting lung. However, the increase was only significant in kidney and bone. It must be recalled that Cr is a highly toxic metal, being one of the chemical forms (Cr^6+^) fully recognized as carcinogenic [23,24]. On the other hand, the mean Pb levels in autopsy tissues were significantly lower in 2019 than in 1998, with the only exception of kidney, an organ where Pb could not be detected in the baseline survey. This fact would be closely related to the banning of Pb as a gasoline additive, introduced in the early 2000s [25].

**Figure 1:**
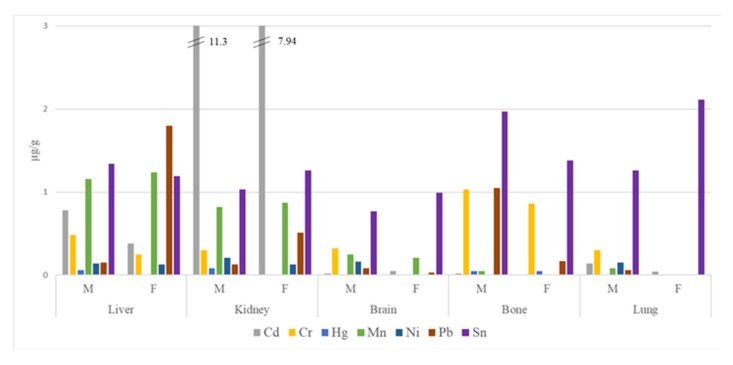
Concentrations (in µg/g) of trace elements in samples of autopsy tissues collected in 2019 according to the sex of the subjects (M: male; F: female).

The concentrations of trace elements in five autopsy tissues according to sex are depicted in Figure 1. Unfortunately, a proper statistical study could not be performed, since 19 out of the 20 subjects were men. Therefore, scientifically valid conclusions cannot be extracted from such a poorly representative number of samples. Anyway, the most notable finding is that the only recruited woman had higher levels of Pb than the mean of men in liver (1.80 vs. 0.15 µg/g) and kidney (0.51 vs. 0.13 µg/g), while in the other tissues, Pb concentrations were higher in men. In addition, the levels of Cr were higher in the tissue samples corresponding to men than those from women.

Table 3 presents the mean concentrations of the trace elements analyzed in autopsy tissues according to age. Three age groups were considered: <35 (*n* = 3), 35–65 (*n* = 10), and >65 (*n* = 7). Due to the relatively small number of samples, a statistical comparison among groups could not be conducted. However, the data may be indicative to set age-specific trends. A correlation study of the metal concentrations in the autopsy tissues was also performed, taking into account that each subject provided five different tissues (brain, bone, kidney, liver, and lung). Notwithstanding, none of the individuals showed a general increase in the body burdens of trace elements, as Pearson correlations among tissues were not statistically significant.

## 4. Discussion

In recent years, a number of scientific studies on the concentrations of metals in humans—for occupationally and non-occupationally exposed populations—have been published [26]. Recently, Dudek-Adamska et al. [27] analyzed the concentrations of Cr in samples of blood and internal organs collected at autopsy from 21 female and 39 male non-occupationally exposed subjects in Southern Poland. Reference ranges of Cr in brain, liver, kidney and lung were 4.7–136, 11–506, 2.9–298 and 13–798 ng/g, respectively. The concentrations corresponding to individuals living near the HWI of Constantí would be within that range, but in the upper side for liver, kidney and lung. In turn, the mean levels of Cr in brain for the population of the current study was 2-fold higher than for the Polish residents. In Sweden, Akerstrom et al. [28] studied the relationship between Hg in kidney, blood, and urine in environmentally exposed individuals, and its implications for biomonitoring. The mean concentration of Hg in kidney from 152 healthy kidney donors (65 men and 87 women) was reported to be 0.33 µg/g, a value 4-times higher than that observed in the autopsied subjects who had been living near the HWI here assessed.

The analysis of trace elements in autopsy tissues is part of a large surveillance program on the HWI. In 2017, the concentrations of the same elements in samples of human hair from schoolchildren and of blood from an adult population, all of them living near the facility, were determined [18,19]. The temporal decrease of Pb in autopsy tissues was also observed in the other biomonitors. In 2017, the mean concentration of Pb in human hair from schoolchildren was 1.44 µg/g, being significantly lower than the value found in the baseline survey (5.81 µg/g). In blood, the mean Pb level in 2017 was 12.98 µg/kg, with a significant (*p* < 0.05) reduction with respect to the previous (2012) study but non-significant (*p* > 0.05) compared to the baseline survey. In addition, the notable increase of Cr observed in some autopsy tissues was also detected in blood [19], where values increased from undetected levels to 6.29 µg/kg. In contrast, Cr levels in human hair did not increase through time [18]. Since the biological concentrations of environmental contaminants in the human body are highly dependent on the dietary intake, food levels of the same pollutants are also periodically monitored. In our last study, corresponding to data on foodstuffs samples collected in 2013 [29], the estimated dietary intake of Cr and Hg was found to progressively and significantly increase with respect to the baseline study.

Some toxic habits, such as smoking or alcohol consumption, have been pointed out as potential sources of toxic elements (i.e., Cd, Pb) [20], whose exposure is related to adverse health effects, including the probability to develop cancer [30]. In the present study, a correlation between smoking and the burdens of the toxic elements analyzed in the five human tissues was not found. However, the reduced number of samples (n = 5 smokers out of 20 subjects) makes difficult to establish any conclusion in this sense.

## 5. Conclusions

The analysis of the temporal trends of the concentrations of a number of trace elements in five autopsy tissues indicates that there have been fluctuations through time, when comparing the results of the campaign performed in 2019 with those corresponding to the baseline survey (1998). Furthermore, no significant changes were noted between 2013 and 2018 for most elements. A general increasing or decreasing tendency was not found, with the only exceptions of Pb and Cr. On one hand, the mean blood levels of Pb were significantly reduced compared to the baseline (1998) study, mainly due to the effect of banning Pb as a gasoline additive, introduced in the early 2000’s. On the other hand, the average Cr concentration in most tissues is still higher than that found in the baseline study, although the difference was only significant in kidney and bone. In contrast, the 5 analyzed autopsy tissues showed significantly lower concentrations than the levels found in the previous campaign (2013), when a generalized increase, not only in autopsy tissues, but also in some environmental samples, was noticed. That rise was also supported by the increase in the dietary intake of Cr estimated for the adult population living near the HWI.

In any case, the levels of trace elements obtained in the present study are similar to those reported recently in various studies of different countries. The global analysis of the data clearly indicates that air emissions of the HWI have not a significant exposure or accumulation of these elements in individuals living in the area near the facility. Moreover, the temporal changes may be more directly related to differences in the dietary exposure of the population.

## Figures and Tables

**Table 1:** Concentration (in µg/g) of trace elements in samples of autopsy tissues collected in 2019 from subjects who had been living near the HWI of Constantí.

Tissue		Mean	±	St. Dev.	Median	Min	Max	Detection Rate (%)
**LIVER**	**As**		<0.10		−	ND	ND	0
	**Be**		<0.10		−	ND	ND	0
	**Cd**	0.76	±	0.61	0.64	0.13	2.77	100
	**Cr**	0.47	±	0.17	0.54	<0.50	0.74	65
	**Hg**	0.06	±	0.04	0.05	<0.10	0.23	5
	**Mn**	1.16	±	0.28	1.18	0.62	1.83	100
	**Ni**	0.14	±	0.06	0.13	<0.25	0.35	10
	**Pb**	0.23	±	0.42	0.08	<0.025	1.80	80
	**Sn**	1.33	±	0.52	1.21	0.88	3.30	100
	**Tl**		<0.025		−	ND	ND	0
	**V**		<0.50		−	ND	ND	0
**KIDNEY**	**As**		<0.10		−	ND	ND	0
	**Be**		<0.10		−	ND	ND	0
	**Cd**	11.10	±	8.17	8.06	0.83	44.35	100
	**Cr**	0.29	±	0.11	0.25	<0.50	0.58	15
	**Hg**	0.08	±	0.09	0.05	<0.10	0.34	15
	**Mn**	0.82	±	0.23	0.87	0.32	1.22	100
	**Ni**	0.21	±	0.23	0.13	<0.25	1.08	15
	**Pb**	0.15	±	0.17	0.09	<0.025	0.65	85
	**Sn**	1.04	±	0.28	1.08	0.70	1.94	100
	**Tl**		<0.025		−	ND	ND	0
	**V**		<0.50		−	ND	ND	0
**BRAIN**	**As**		<0.10		−	ND	ND	0
	**Be**		<0.10		−	ND	ND	0
	**Cd**	0.02	±	0.02	0.01	<0.025	0.06	30
	**Cr**	0.32	±	0.12	0.25	<0.50	0.58	25
	**Hg**		<0.10		−	ND	ND	0
	**Mn**	0.25	±	0.15	0.21	0.16	0.86	100
	**Ni**	0.16	±	0.11	0.13	<0.25	0.58	10
	**Pb**	0.08	±	0.12	0.02	<0.025	0.47	50
	**Sn**	0.78	±	0.25	0.72	0.54	1.60	100
	**Tl**		<0.025		−	ND	ND	0
	**V**		<0.50		−	ND	ND	0
**BONE**	**As**		<0.10		−	ND	ND	0
	**Be**		<0.10		−	ND	ND	0
	**Cd**	0.02	±	0.02	0.01	<0.025	0.07	30
	**Cr**	1.02	±	0.24	1.00	0.63	1.52	100
	**Hg**		<0.10		−	ND	ND	0
	**Mn**	0.05	±	0.04	0.03	<0.05	0.16	40
	**Ni**		<0.25		−	ND	ND	0
	**Pb**	1.00	±	1.33	0.54	<0.025	5.39	95
	**Sn**	1.94	±	0.64	1.73	1.07	3.51	100
	**Tl**		<0.025		−	ND	ND	0
	**V**		<0.50		−	ND	ND	0
**LUNG**	**As**		<0.10		−	ND	ND	0
	**Be**		<0.10		−	ND	ND	0
	**Cd**	0.13	±	0.18	0.04	<0.025	0.62	65
	**Cr**	0.30	±	0.12	0.25	<0.50	0.62	15
	**Hg**		<0.10		−	ND	ND	0
	**Mn**	0.08	±	0.04	0.07	<0.05	0.15	75
	**Ni**	0.15	±	0.07	0.13	<0.25	0.40	10
	**Pb**	0.05	±	0.11	0.01	<0.025	0.42	35
	**Sn**	1.31	±	0.33	1.20	0.91	2.11	100
	**Tl**		<0.025		−	ND	ND	0
	**V**		<0.50		−	ND	ND	0

**Table 2:** Mean concentration (µg/g) of trace elements in autopsy tissues collected between 1998 and 2019. Temporal trends.

Tissue							% Variation	
		1998	2003	2007	2013	2019	1998–2019	2013–2019
**LIVER**	**As**	<0.05	<0.05	0.07	<0.05	<0.10	-	-
	**Be**	<0.02	<0.05	<0.03	<0.05	<0.10	-	-
	**Cd**	0.95	1.36	0.8	1.38	0.76	−20 ***	−45 **
	**Cr**	0.26	<0.25	0.63	0.66	0.47	81	−29
	**Hg**	0.2	0.14	0.14	<0.05	0.06	−70 *	-
	**Mn**	1.28	1.07	0.99	1.45	1.16	−9	−20
	**Ni**	0.09	<0.1	0.07	<0.10	0.14	56	-
	**Pb**	2.56	0.3	0.35	0.18	0.23	−91 ***	28
	**Sn**	5.06	0.19	0.07	<0.05	1.33	−74	-
	**Tl**	<0.02	<0.01	<0.01	<0.03	<0.025	-	-
	**V**	<0.12	<0.25	<0.25	<0.10	<0.50	-	-
**KIDNEY**	**As**	<0.05	<0.05	0.06	<0.05	<0.10	-	-
	**Be**	<0.02	<0.05	<0.03	<0.05	<0.10	-	-
	**Cd**	17.52	17.46	14.72	21.15	11.1	−37	−48
	**Cr**	0.09	<0.25	0.42	0.66	0.29	222 ***	−56 **
	**Hg**	0.33	0.23	0.3	0.15	0.08	−76 *	−47 *
	**Mn**	1.01	0.74	0.78	1.09	0.82	−19	−25
	**Ni**	<0.01	<0.10	<0.05	<0.10	0.21	-	-
	**Pb**	<0.02	0.06	0.77	0.1	0.15	-	50
	**Sn**	1.66	0.17	0.05	<0.05	1.04	−37 *	-
	**Tl**	<0.02	<0.01	<0.01	<0.03	<0.025	-	-
	**V**	<0.12	<0.25	<0.25	<0.10	<0.50	-	-
**BRAIN**	**As**	<0.05	<0.05	<0.05	<0.05	<0.10	-	-
	**Be**	<0.02	<0.05	<0.03	<0.05	<0.10	-	-
	**Cd**	0.03	0.02	0.32	<0.05	0.02	−33	-
	**Cr**	0.22	<0.25	0.45	0.57	0.32	45	−44
	**Hg**	<0.05	<0.05	0.1	<0.05	<0.10	-	-
	**Mn**	0.22	0.03	0.24	0.33	0.25	14	−24
	**Ni**	<0.01	<0.10	0.36	<0.05	0.16	-	-
	**Pb**	1.41	0.06	0.1	<0.05	0.08	−94 ***	-
	**Sn**	1.32	0.09	0.03	<0.05	0.78	−41	-
	**Tl**	<0.02	<0.01	<0.01	<0.05	<0.025	-	-
	**V**	<0.12	<0.25	0.28	<0.05	<0.50	-	-
**BONE**	**As**	0.06	<0.05	0.19	<0.05	<0.10	-	-
	**Be**	<0.02	<0.05	0.03	<0.05	<0.10	-	-
	**Cd**	0.04	0.05	0.04	<0.03	0.02	−50 **	-
	**Cr**	0.51	<0.25	1.39	1.38	1.02	100 ***	−26
	**Hg**	<0.05	<0.05	0.05	<0.05	<0.10	-	-
	**Mn**	0.06	<0.03	0.25	0.13	0.05	−17	−62 *
	**Ni**	0.64	1.16	1.53	<0.10	<0.25	-	-
	**Pb**	3.99	2.11	2.66	1.39	1	−75 ***	−28
	**Sn**	7.4	0.34	0.31	0.17	1.94	−74 ***	1041 ***
	**Tl**	<0.02	<0.01	<0.01	<0.03	<0.025	-	-
	**V**	<0.12	<0.25	<0.25	<0.10	<0.50	-	-
**LUNG**	**As**	<0.05	<0.05	0.14	<0.05	<0.10	-	-
	**Be**	<0.02	<0.05	<0.03	<0.05	<0.10	-	-
	**Cd**	0.42	0.18	0.27	0.26	0.13	−69	−50
	**Cr**	0.33	0.25	0.58	0.64	0.3	−9	−53 *
	**Hg**	<0.05	<0.05	<0.05	<0.05	<0.10	-	-
	**Mn**	0.13	0.04	0.3	0.21	0.08	−38	−62 **
	**Ni**	0.08	0.12	0.07	<0.10	0.15	88 **	-
	**Pb**	2.27	0.13	0.08	0.05	0.05	−98 ***	0
	**Sn**	2.16	0.2	0.07	<0.05	1.31	−39	-
	**Tl**	<0.02	<0.01	<0.01	<0.03	<0.025	-	-
	**V**	<0.12	<0.25	0.58	<0.10	<0.50	-	-

* *p* < 0.05; ** *p* < 0.01; *** *p* < 0.001.

**Table 3:** Concentrations (µg/g) of trace elements in samples of autopsy tissues collected in 2019 according to the age of the subjects.

Tissue		<35 years (*n* = 3)			35–65 years (*n* = 10)			>65 years (*n* = 7)		
		Mean	±	St. Dev.	Mean	±	St. Dev.	Mean	±	St. Dev.
**LIVER**	**As**		<0.10			<0.10			<0.10	
	**Be**		<0.10			<0.10			<0.10	
	**Cd**	0.99	±	0.53	0.55	±	0.34	0.96	±	0.86
	**Cr**	0.47	±	0.19	0.46	±	0.19	0.48	±	0.16
	**Hg**		<0.10			<0.10			0.08	
	**Mn**	1.32	±	0.45	1.06	±	0.24	1.24	±	0.25
	**Ni**	0.13	±	0.00	0.14	±	0.05	0.16	±	0.08
	**Pb**	0.23	±	0.28	0.33	±	0.58	0.08	±	0.09
	**Sn**	2.03	±	1.20	1.20	±	0.30	1.21	±	0.11
	**Tl**		<0.025			<0.025			<0.025	
	**V**		<0.50			<0.50			<0.50	
**KIDNEY**	**As**		<0.10			<0.10			<0.10	
	**Be**		<0.10			<0.10			<0.10	
	**Cd**	5.50	±	3.54	12.98	±	8.99	10.81	±	7.94
	**Cr**	0.33	±	0.15	0.28	±	0.10	0.29	±	0.11
	**Hg**	0.15	±	0.17	0.08	±	0.09		<0.10	
	**Mn**	1.04	±	0.34	0.84	±	0.20	0.69	±	0.22
	**Ni**		<0.25		0.25	±	0.31	0.18	±	0.14
	**Pb**	0.06	±	0.10	0.20	±	0.22	0.11	±	0.08
	**Sn**	0.85	±	0.18	1.15	±	0.31	0.96	±	0.22
	**Tl**		<0.025			<0.025			<0.025	
	**V**		<0.50			<0.50			<0.50	
**BRAIN**	**As**		<0.10			<0.10			<0.10	
	**Be**		<0.10			<0.10			<0.10	
	**Cd**	0.03	±	0.02	0.02	±	0.02	0.02	±	0.01
	**Cr**	0.34	±	0.15	0.33	±	0.14	0.29	±	0.10
	**Hg**		<0.10			<0.10			<0.10	
	**Mn**	0.22	±	0.05	0.29	±	0.20	0.21	±	0.03
	**Ni**	0.13	±	0.00	0.17	±	0.14	0.16	±	0.09
	**Pb**	0.11	±	0.11	0.05	±	0.04	0.12	±	0.19
	**Sn**	0.73	±	0.10	0.79	±	0.32	0.80	±	0.17
	**Tl**		<0.025			<0.025			<0.025	
	**V**		<0.50			<0.50			<0.50	
**LUNG**	**As**		<0.10			<0.10			<0.10	
	**Be**		<0.10			<0.10			<0.10	
	**Cd**	0.10	±	0.16	0.16	±	0.21	0.10	±	0.14
	**Cr**	0.37	±	0.21	0.28	±	0.08	0.30	±	0.14
	**Hg**		<0.10			<0.10			<0.10	
	**Mn**	0.10	±	0.03	0.07	±	0.05	0.08	±	0.04
	**Ni**		<0.25		0.15	±	0.09	0.15	±	0.06
	**Pb**		<0.025		0.06	±	0.13	0.07	±	0.10
	**Sn**	1.30	±	0.30	1.26	±	0.33	1.38	±	0.36
	**Tl**		<0.025			<0.025			<0.025	
	**V**		<0.50			<0.50			<0.50	
**BONE**	**As**		<0.10			<0.10			<0.10	
	**Be**		<0.10			<0.10			<0.10	
	**Cd**		<0.025		0.03	±	0.02	0.02	±	0.01
	**Cr**	0.70	±	0.06	1.06	±	0.31	1.11	±	0.12
	**Hg**		<0.10			<0.10			<0.10	
	**Mn**	0.05	±	0.02	0.04	±	0.07	0.06	±	0.05
	**Ni**	0.13	±	0.00	0.13	±	0.00	0.13	±	0.00
	**Pb**	0.06	±	0.07	0.43	±	0.34	2.23	±	1.69
	**Sn**	2.39	±	0.69	1.80	±	0.36	1.94	±	0.61
	**Tl**		<0.025			<0.025			<0.025	
	**V**		<0.50			<0.50			<0.50	
